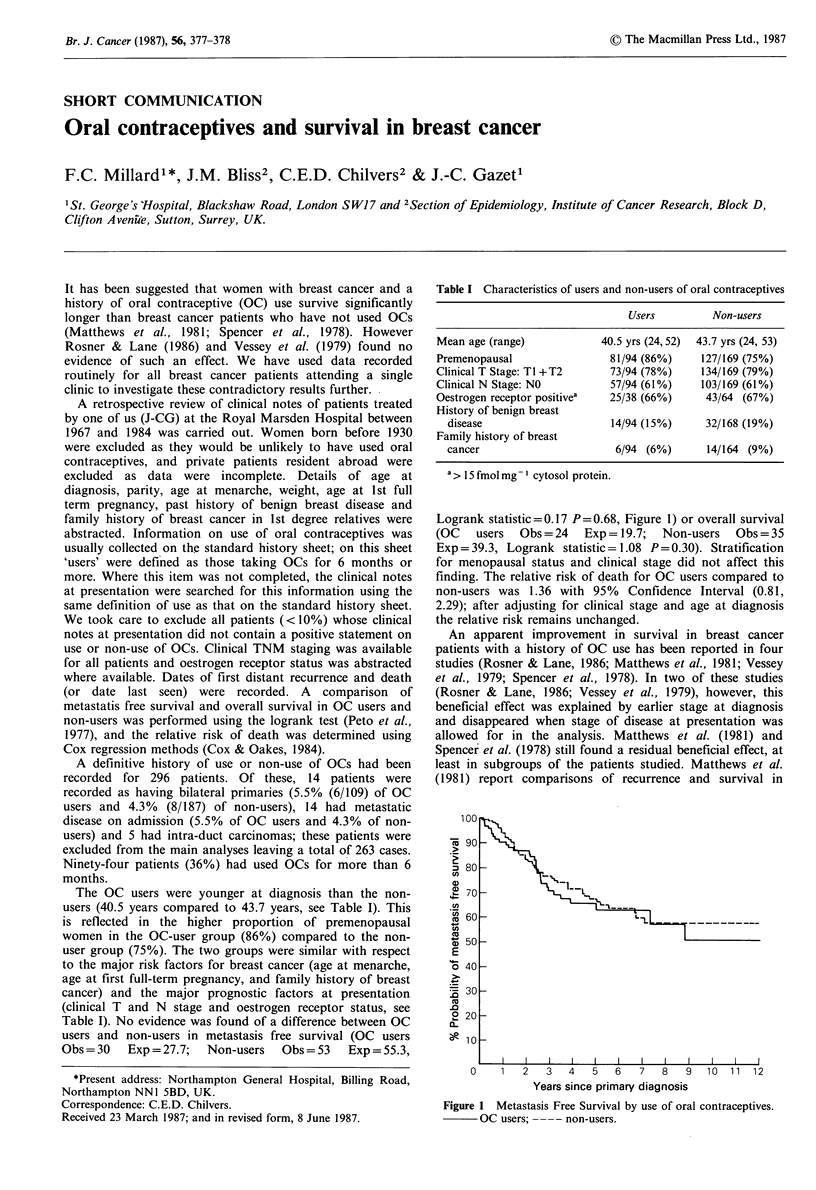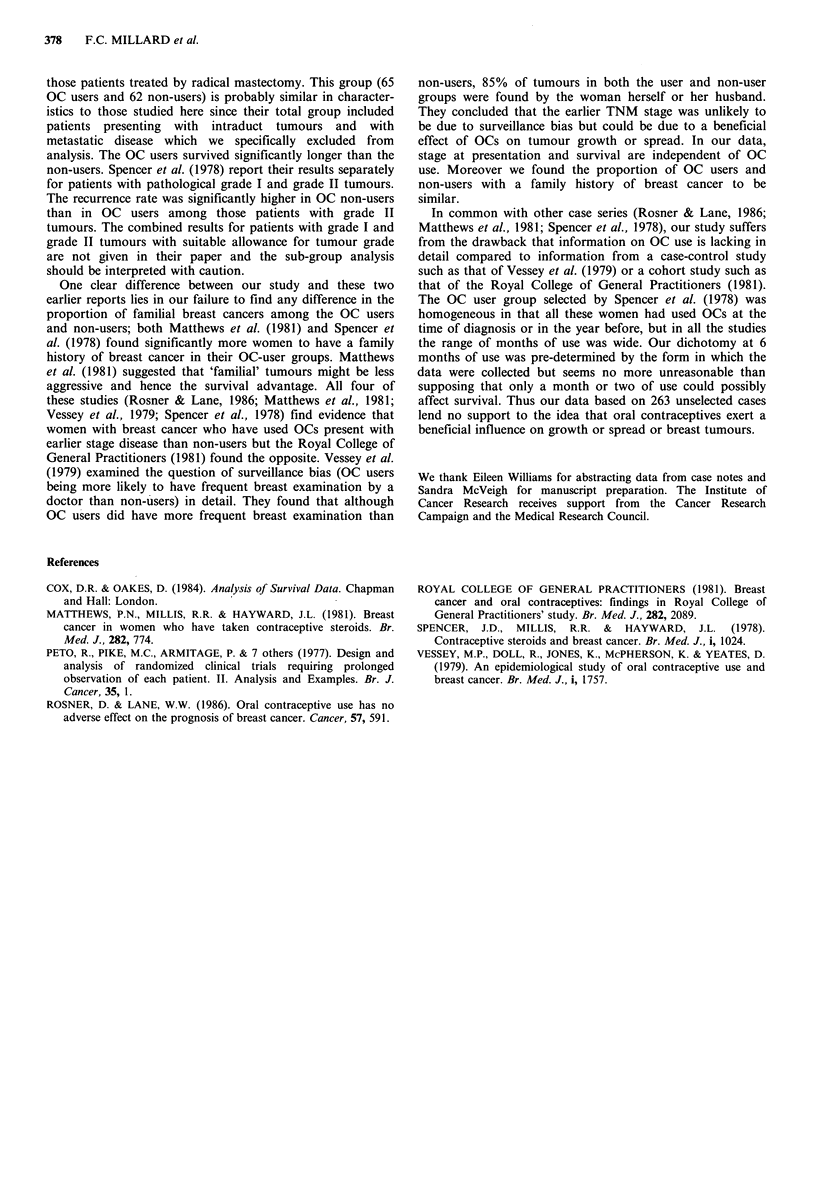# Oral contraceptives and survival in breast cancer.

**DOI:** 10.1038/bjc.1987.206

**Published:** 1987-09

**Authors:** F. C. Millard, J. M. Bliss, C. E. Chilvers, J. C. Gazet

**Affiliations:** St. George's Hospital, London, UK.


*Br. J. Cancer* (1987), **56**, 377–378 © The Macmillan Press Ltd., 1987

SHORT COMMUNICATION

### Oral contraceptives and survival in breast cancer

F.C. Millard[1]*, J.M. Bliss[2], C.E.D. Chilvers[2] & J.-C. Gazet[1]

[1]*St. George's Hospital, Blackshaw Road, London SW17 and* [2]*Section of Epidemiology, Institute of Cancer Research, Block D, Clifton Avenue, Sutton, Surrey, UK.*

It has been suggested that women with breast cancer and a history of oral contraceptive (OC) use survive significantly longer than breast cancer patients who have not used OCs (Matthews *et al.*, 1981; Spencer *et al.*, 1978). However Rosner & Lane (1986) and Vessey *et al.* (1979) found no evidence of such an effect. We have used data recorded routinely for all breast cancer patients attending a single clinic to investigate these contradictory results further.

A retrospective review of clinical notes of patients treated by one of us (J-CG) at the Royal Marsden Hospital between 1967 and 1984 was carried out. Women born before 1930 were excluded as they would be unlikely to have used oral contraceptives, and private patients resident abroad were excluded as data were incomplete. Details of age at diagnosis, parity, age at menarche, weight, age at 1st full term pregnancy, past history of benign breast disease and family history of breast cancer in 1st degree relatives were abstracted. Information on use of oral contraceptives was usually collected on the standard history sheet; on this sheet 'users' were defined as those taking OCs for 6 months or more. Where this item was not completed, the clinical notes at presentation were searched for this information using the same definition of use as that on the standard history sheet. We took care to exclude all patients (<10%) whose clinical notes at presentation did not contain a positive statement on use or non-use of OCs. Clinical TNM staging was available for all patients and oestrogen receptor status was abstracted where available. Dates of first distant recurrence and death (or date last seen) were recorded. A comparison of metastatis free survival and overall survival in OC users and non-users was performed using the logrank test (Peto *et al.*, 1977), and the relative risk of death was determined using Cox regression methods (Cox & Oakes, 1984).

A definitive history of use or non-use of OCs had been recorded for 296 patients. Of these, 14 patients were recorded as having bilateral primaries (5.5% (6/109) of OC users and 4.3% (8/187) of non-users), 14 had metastatic disease on admission (5.5% of OC users and 4.3% of non-users) and 5 had intra-duct carcinomas; these patients were excluded from the main analyses leaving a total of 263 cases. Ninety-four patients (36%) had used OCs for more than 6 months.

The OC users were younger at diagnosis than the non-users (40.5 years compared to 43.7 years, see Table I). This is reflected in the higher proportion of premenopausal women in the OC-user group (86%) compared to the non-user group (75%). The two groups were similar with respect to the major risk factors for breast cancer (age at menarche, age at first full-term pregnancy, and family history of breast cancer) and the major prognostic factors at presentation (clinical T and N stage and oestrogen receptor status, see Table I). No evidence was found of a difference between OC users and non-users in metastasis free survival (OC users Obs = 30 Exp = 27.7; Non-users Obs = 53 Exp = 55.3, Logrank statistic = 0.17 $P$ = 0.68, Figure 1) or overall survival (OC users Obs = 24 Exp = 19.7; Non-users Obs = 35 Exp = 39.3, Logrank statistic = 1.08 $P$ = 0.30). Stratification for menopausal status and clinical stage did not affect this finding. The relative risk of death for OC users compared to non-users was 1.36 with 95% Confidence Interval (0.81, 2.29); after adjusting for clinical stage and age at diagnosis the relative risk remains unchanged.

**Table I** Characteristics of users and non-users of oral contraceptives

| | *Users* | *Non-users* |
|---|---|---|
| Mean age (range) | 40.5 yrs (24, 52) | 43.7 yrs (24, 53) |
| Premenopausal | 81/94 (86%) | 127/169 (75%) |
| Clinical T Stage: T1 + T2 | 73/94 (78%) | 134/169 (79%) |
| Clinical N Stage: N0 | 57/94 (61%) | 103/169 (61%) |
| Oestrogen receptor positive[a] | 25/38 (66%) | 43/64 (67%) |
| History of benign breast disease | 14/94 (15%) | 32/168 (19%) |
| Family history of breast cancer | 6/94 (6%) | 14/164 (9%) |

[a] > 15 fmol mg$^{-1}$ cytosol protein.

An apparent improvement in survival in breast cancer patients with a history of OC use has been reported in four studies (Rosner & Lane, 1986; Matthews *et al.*, 1981; Vessey *et al.*, 1979; Spencer *et al.*, 1978). In two of these studies (Rosner & Lane, 1986; Vessey *et al.*, 1979), however, this beneficial effect was explained by earlier stage at diagnosis and disappeared when stage of disease at presentation was allowed for in the analysis. Matthews *et al.* (1981) and Spencer *et al.* (1978) still found a residual beneficial effect, at least in subgroups of the patients studied. Matthews *et al.* (1981) report comparisons of recurrence and survival in

**Figure 1** Metastasis Free Survival by use of oral contraceptives. ——— OC users; - - - - non-users.

*Present address: Northampton General Hospital, Billing Road, Northampton NN1 5BD, UK.

Correspondence: C.E.D. Chilvers.

Received 23 March 1987; and in revised form, 8 June 1987.

those patients treated by radical mastectomy. This group (65 OC users and 62 non-users) is probably similar in characteristics to those studied here since their total group included patients presenting with intraduct tumours and with metastatic disease which we specifically excluded from analysis. The OC users survived significantly longer than the non-users. Spencer *et al.* (1978) report their results separately for patients with pathological grade I and grade II tumours. The recurrence rate was significantly higher in OC non-users than in OC users among those patients with grade II tumours. The combined results for patients with grade I and grade II tumours with suitable allowance for tumour grade are not given in their paper and the sub-group analysis should be interpreted with caution.

One clear difference between our study and these two earlier reports lies in our failure to find any difference in the proportion of familial breast cancers among the OC users and non-users; both Matthews *et al.* (1981) and Spencer *et al.* (1978) found significantly more women to have a family history of breast cancer in their OC-user groups. Matthews *et al.* (1981) suggested that 'familial' tumours might be less aggressive and hence the survival advantage. All four of these studies (Rosner & Lane, 1986; Matthews *et al.*, 1981; Vessey *et al.*, 1979; Spencer *et al.*, 1978) find evidence that women with breast cancer who have used OCs present with earlier stage disease than non-users but the Royal College of General Practitioners (1981) found the opposite. Vessey *et al.* (1979) examined the question of surveillance bias (OC users being more likely to have frequent breast examination by a doctor than non-users) in detail. They found that although OC users did have more frequent breast examination than non-users, 85% of tumours in both the user and non-user groups were found by the woman herself or her husband. They concluded that the earlier TNM stage was unlikely to be due to surveillance bias but could be due to a beneficial effect of OCs on tumour growth or spread. In our data, stage at presentation and survival are independent of OC use. Moreover we found the proportion of OC users and non-users with a family history of breast cancer to be similar.

In common with other case series (Rosner & Lane, 1986; Matthews *et al.*, 1981; Spencer *et al.*, 1978), our study suffers from the drawback that information on OC use is lacking in detail compared to information from a case-control study such as that of Vessey *et al.* (1979) or a cohort study such as that of the Royal College of General Practitioners (1981). The OC user group selected by Spencer *et al.* (1978) was homogeneous in that all these women had used OCs at the time of diagnosis or in the year before, but in all the studies the range of months of use was wide. Our dichotomy at 6 months of use was pre-determined by the form in which the data were collected but seems no more unreasonable than supposing that only a month or two of use could possibly affect survival. Thus our data based on 263 unselected cases lend no support to the idea that oral contraceptives exert a beneficial influence on growth or spread or breast tumours.

We thank Eileen Williams for abstracting data from case notes and Sandra McVeigh for manuscript preparation. The Institute of Cancer Research receives support from the Cancer Research Campaign and the Medical Research Council.

### References

COX, D.R. & OAKES, D. (1984). *Analysis of Survival Data*. Chapman and Hall: London.

MATTHEWS, P.N., MILLIS, R.R. & HAYWARD, J.L. (1981). Breast cancer in women who have taken contraceptive steroids. *Br. Med. J.*, **282**, 774.

PETO, R., PIKE, M.C., ARMITAGE, P. & 7 others (1977). Design and analysis of randomized clinical trials requiring prolonged observation of each patient. II. Analysis and Examples. *Br. J. Cancer*, **35**, 1.

ROSNER, D. & LANE, W.W. (1986). Oral contraceptive use has no adverse effect on the prognosis of breast cancer. *Cancer*, **57**, 591.

ROYAL COLLEGE OF GENERAL PRACTITIONERS (1981). Breast cancer and oral contraceptives: findings in Royal College of General Practitioners' study. *Br. Med. J.*, **282**, 2089.

SPENCER, J.D., MILLIS, R.R. & HAYWARD, J.L. (1978). Contraceptive steroids and breast cancer. *Br. Med. J.*, **i**, 1024.

VESSEY, M.P., DOLL, R., JONES, K., McPHERSON, K. & YEATES, D. (1979). An epidemiological study of oral contraceptive use and breast cancer. *Br. Med. J.*, **i**, 1757.